# Supplementary material for: Rapid and efficient ambient temperature X-ray crystal structure determination at Turkish Light Source
Source: Sci Rep. 2023 May 19;13:8123. doi: 10.1038/s41598-023-33989-0 (PMC10198979; doi:10.1038/s41598-023-33989-0)
Supplement: Supplementary file 1 — Supplementary Information. [file 41598_2023_33989_MOESM1_ESM.docx]

**Supplementary Table-1**: Three different run parameters.

| **Sample**  **name** | **Frames/**  **Runs** | **Scan**  **width**  **(degrees)** | **Detector**  **distance**  **(mm)** | **Rotation (degrees)** | **Pixel** | **Max**  **cps** | **Exposure**  **time (s)** | **Dose**  **time** | **Total**  **time** | **Beam slit** | **Sample**  **temperature**  **(K)** | **Hypix**  **temperature**  **(°C)** | **Hypix**  **humidity (%)** |
| --- | --- | --- | --- | --- | --- | --- | --- | --- | --- | --- | --- | --- | --- |
| C11_D1 | 21/1 | 1.00 | 100.00 | (-11.25) - (+11.25) | 0-3728 | 1994 | 5.0 | 1m45s | 1m49s | 1.0 mR divergence, 10% intensity | 299.6 | 42.0 – 46.0 | 5 |
| D11_D1 | 21/1 | 1.00 | 100.00 | (-11.25) - (+11.25) | 0-6552 | 2222 | 5.0 | 1m45s | 1m49s | 1.0 mR divergence, 10% intensity | 299.6 | 42.0 – 46.0 | 5 |
| D12_D1 | 15/1 | 1.00 | 100.00 | (-11.25) - (+11.25) | 0-7334 | 3980 | 5.0 | 1m15s | 1m19s | 1.0 mR divergence, 10% intensity | 299.6 | 42.0 – 46.0 | 5 |


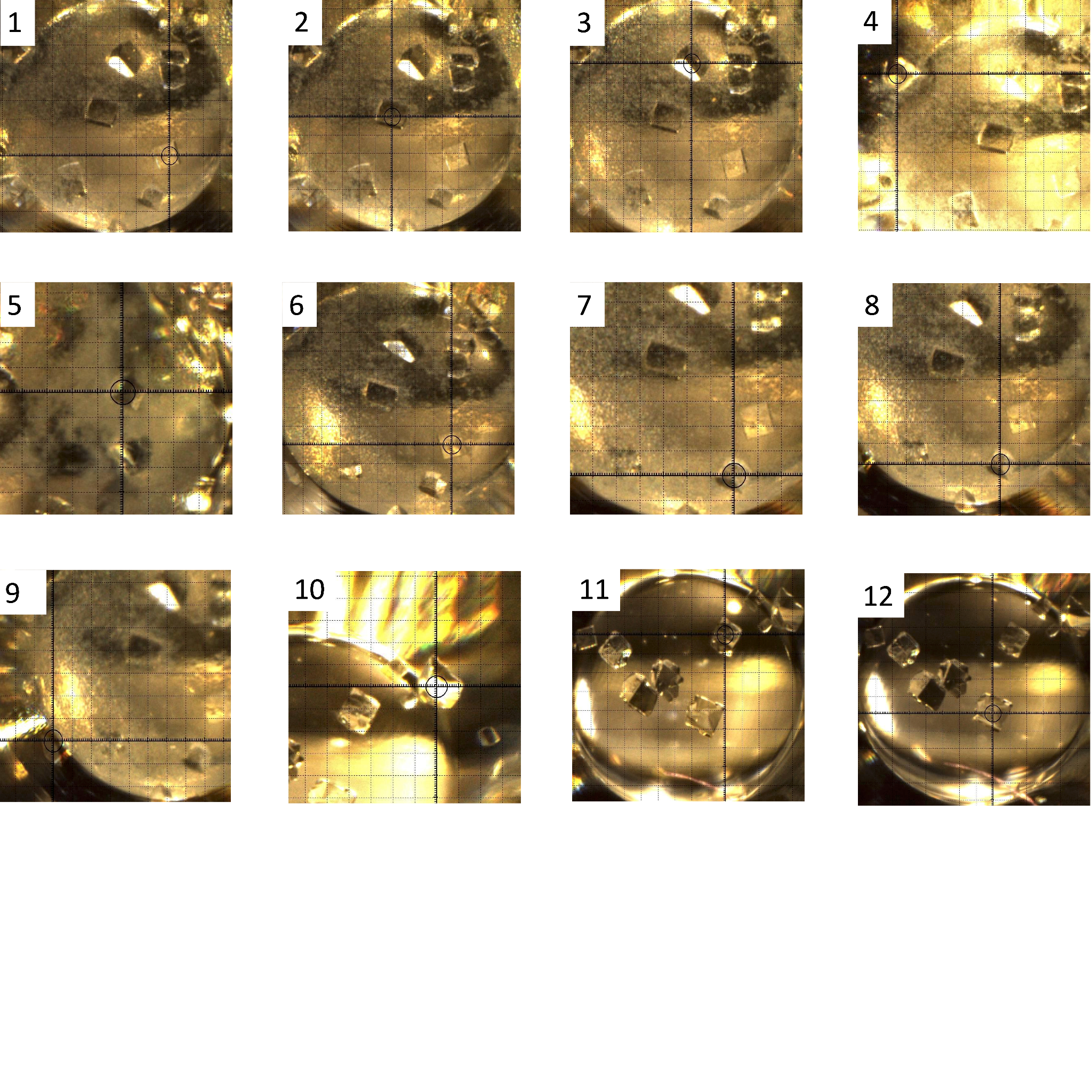


**Supplementary Figure 1.** Multiple crystals for data collection experiments.

**
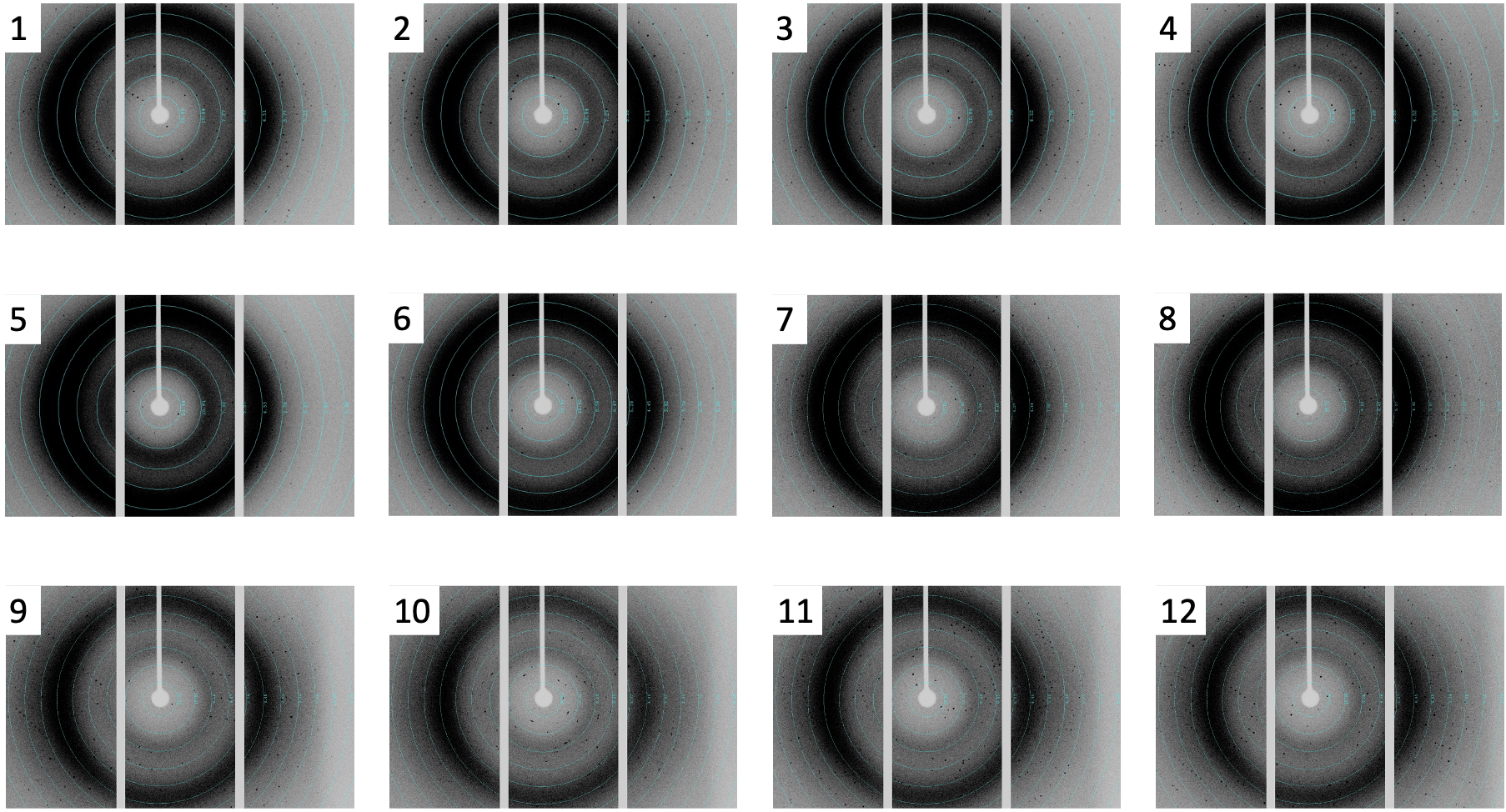
**

**Supplementary Figure 2.** Diffraction patterns collected from each crystal.

**
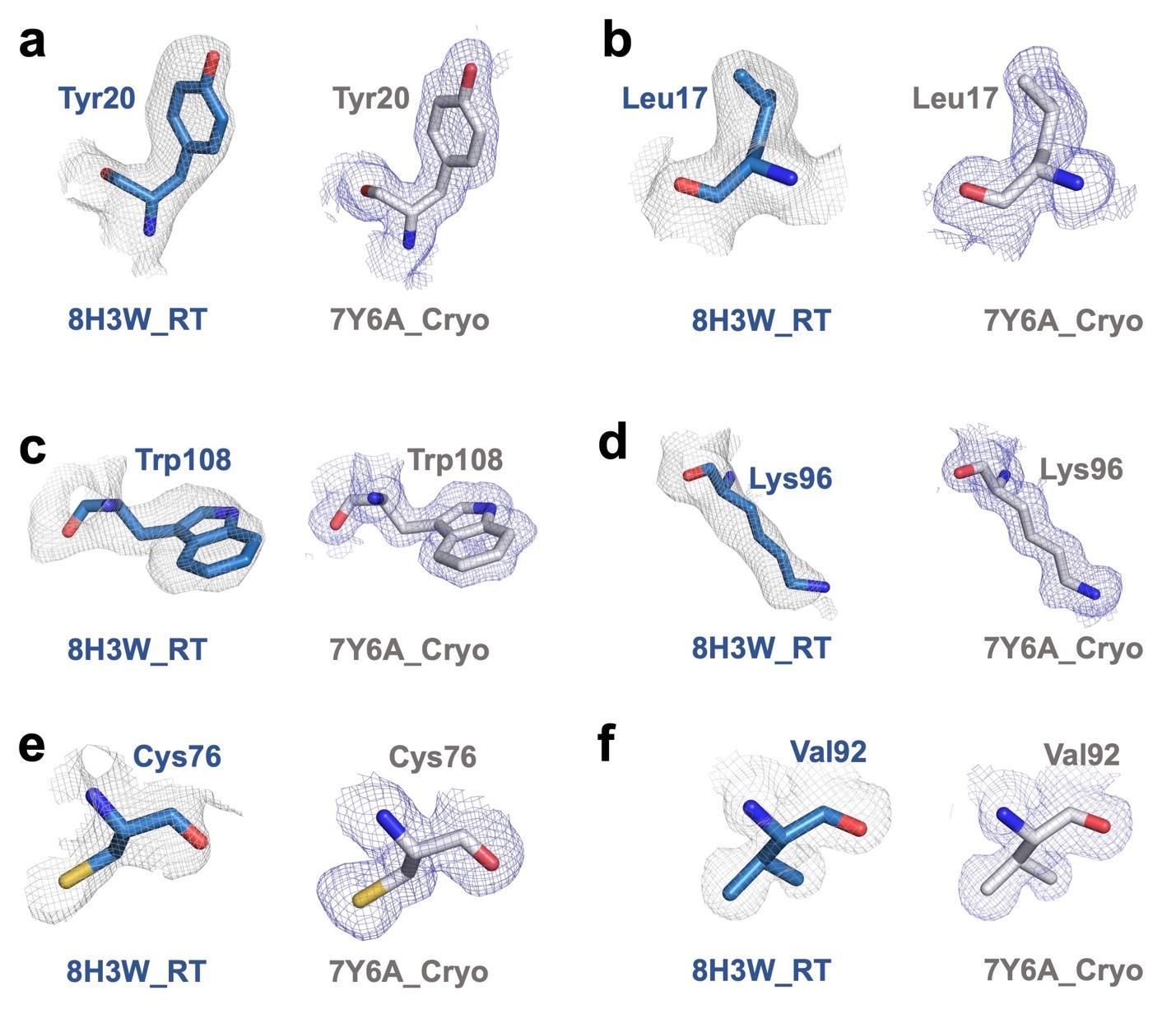
**

**Supplementary Figure 3.** Comparison of electron density map of ambient temperature (PDB ID: 8H3W) and cryogenic temperature (PDB ID: 7Y6A) chicken egg lysozyme structure for residues. 2Fo-Fc simulated annealing-omit map for ambient temperature structure is shown in gray while 2Fo-Fc simulated annealing-omit map for cryogenic structure is shown in slate. Generated with *PyMOL version 2.3* ^27^.


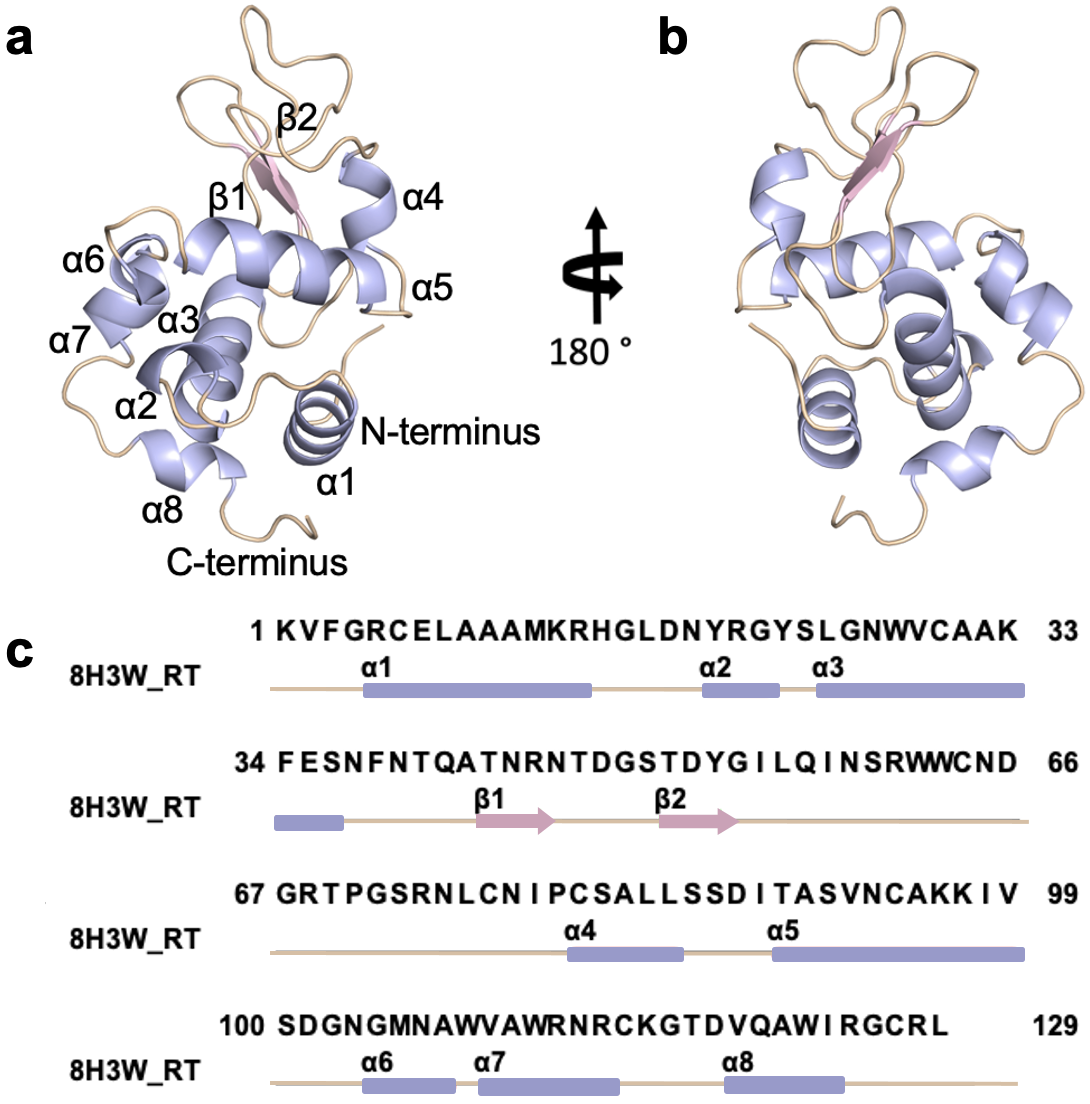


**Supplementary Figure 4.** Secondary structure representation of chicken egg lysozyme at ambient temperature (PDB ID: 8H3W). **(a-b)** The chicken egg lysozyme structure at ambient temperature is shown with cartoon representation. Two side views are presented in the panel by rotating the structure 180 degrees on the y-axis. **(c)** Structure-based sequence alignment of lysozyme is indicated with secondary structures based on color code (alpha-helices: lightblue; beta-sheets: lightpink; loops: wheat). Generated with *PyMOL version 2.3* ^27^.


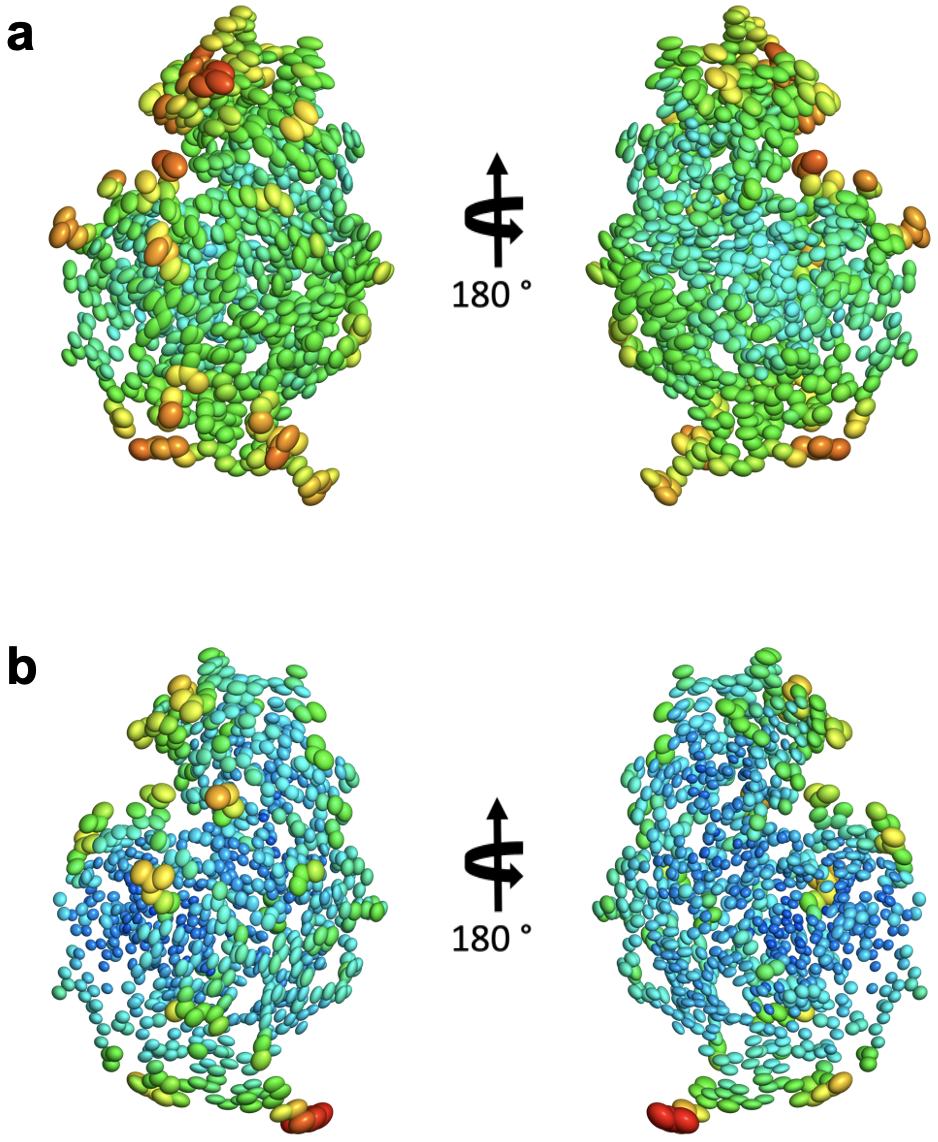


**Supplementary Figure 5.** Ellipsoid presentation of chicken egg lysozyme at **(a)** ambient and **(b)** cryogenic temperature. Generated with *PyMOL version 2.3* ^27^.


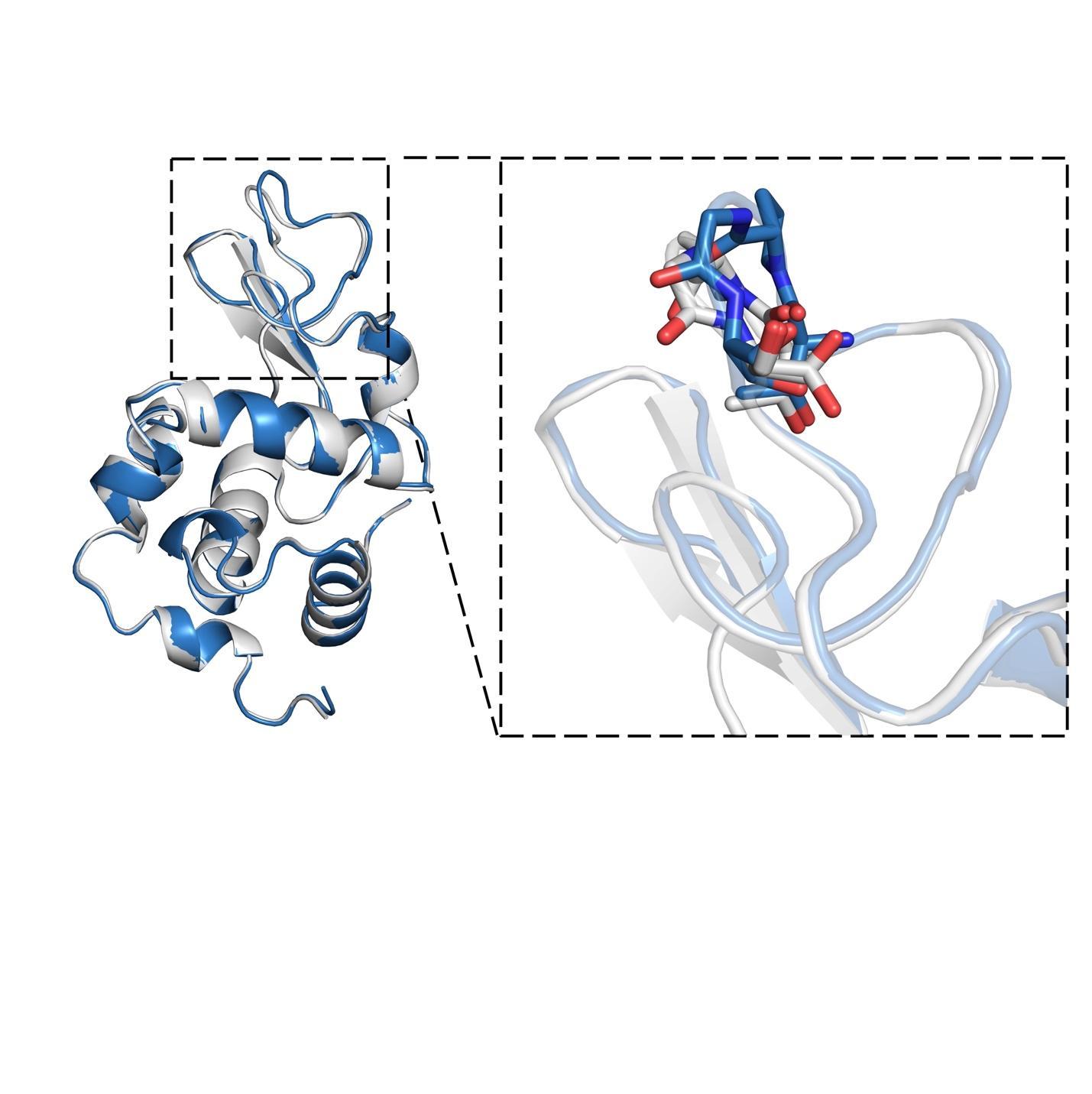


**Supplementary Figure 6.** Conformational changes on loop6. Ambient temperature lysozyme is shown in skyblue. Cryogenic lysozyme is shown in gray. Generated with *PyMOL version 2.3* ^27^.


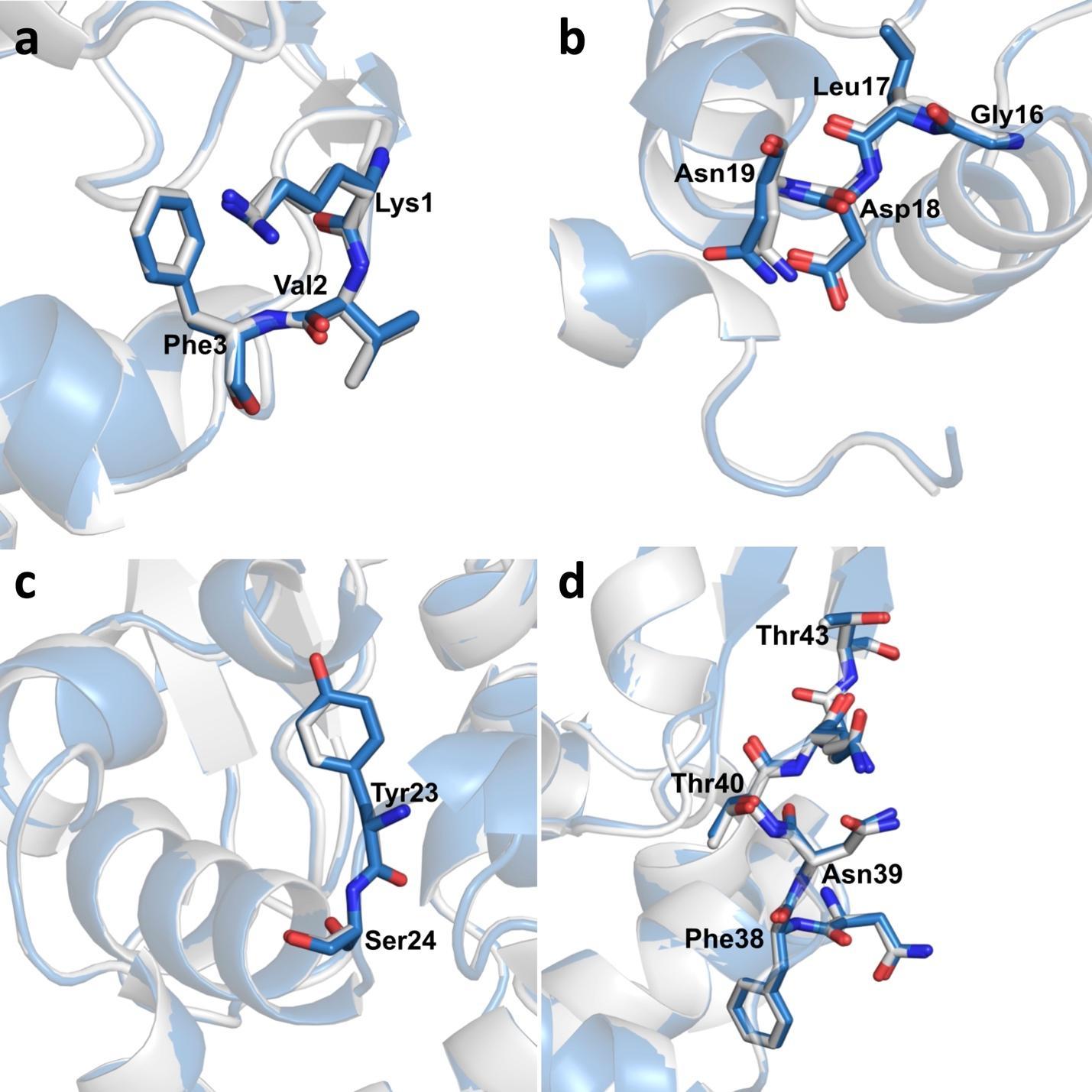


**Supplementary Figure 7.** Loop region (1-4) comparisons of ambient temperature lysozyme (skyblue) with cryogenic lysozyme (gray). RMS values are shown in parentheses. **(a)** Loop 1 (*0.109 Å*); **(b)** Loop 2 (*0.117 Å***)**; **(c)** Loop 3 (*0.001 Å*); **(d)** Loop 4 (*0.091 Å*). Generated with *PyMOL version 2.3* ^27^.


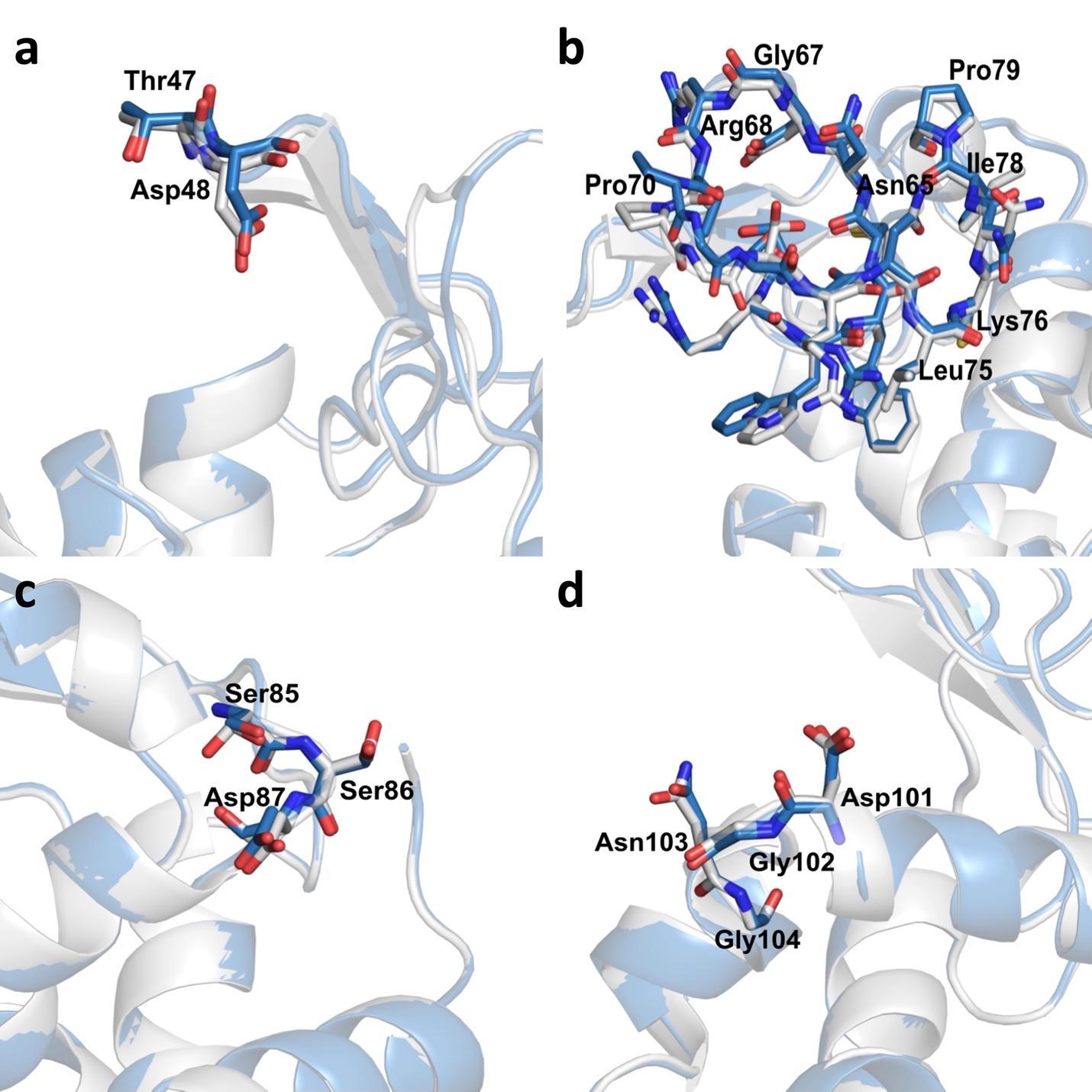


**Supplementary Figure 8.** Loop region (5-8) comparisons of ambient temperature lysozyme (skyblue) with cryogenic lysozyme (gray). RMS values are shown in parentheses. **(a)** Loop 5 (*0.235 Å*); **(b)** Loop 6 (*0.066 Å*); **(c)** Loop 7 (*0.066 Å*); **(d)** Loop 8 (*0.095 Å*). Generated with *PyMOL version 2.3* ^27^.

**
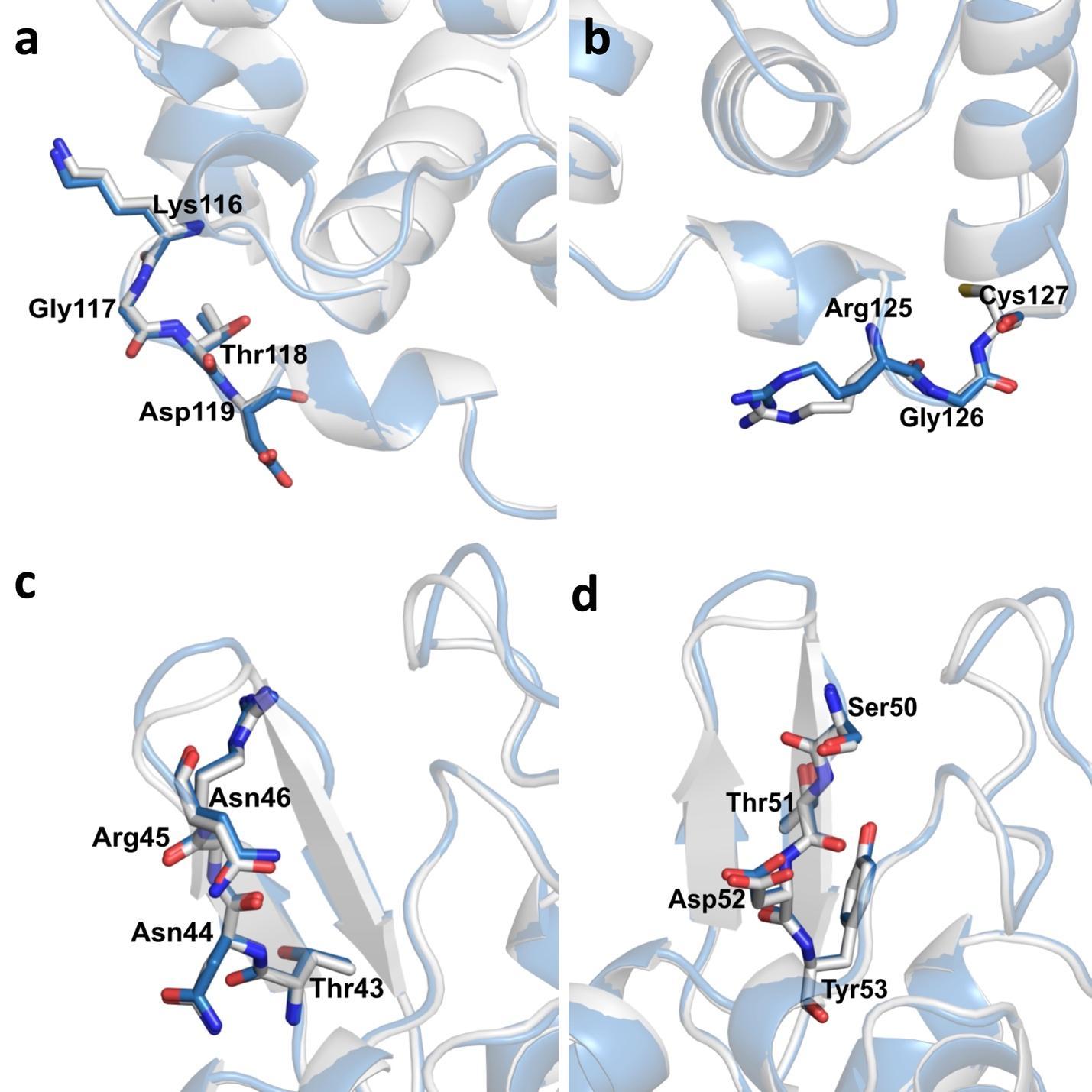
**

**Supplementary Figure 9.** Loop and beta-sheet region comparisons of ambient temperature lysozyme (skyblue) with cryogenic lysozyme (gray). RMS values are shown in parentheses. **(a)** Loop 9 (*0.052 Å*); **(b)** Loop 10 *(0.045 Å*) ; **(c)** Beta sheet 1 (*0.022 Å*) ; **(d)** Beta- sheet 2 (*0.066 Å*). Generated with *PyMOL version 2.3* ^27^.

**
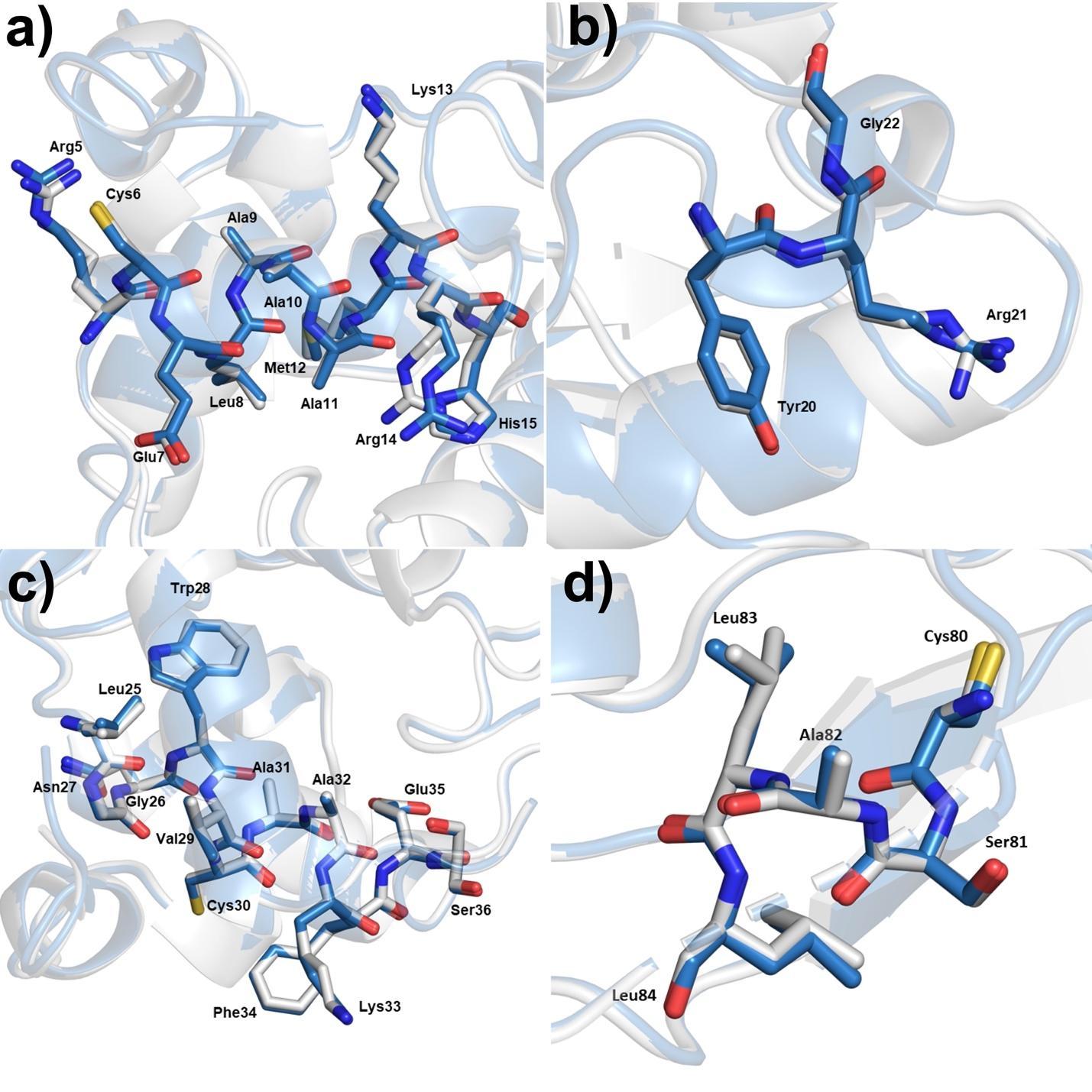
**

**Supplementary Figure 10.** Helix region (1-4) comparisons of ambient temperature lysozyme (skyblue) with cryogenic lysozyme (gray). RMS values are shown in parentheses. **(a)** Helix 1 (*0.097 Å*); **(b)** Helix 2 (*0.092 Å*); **(c)** Helix 3 (*0.070 Å*); **(d)** Helix 4 (*0.061 Å*). Generated with *PyMOL version 2.3* ^27^.

**
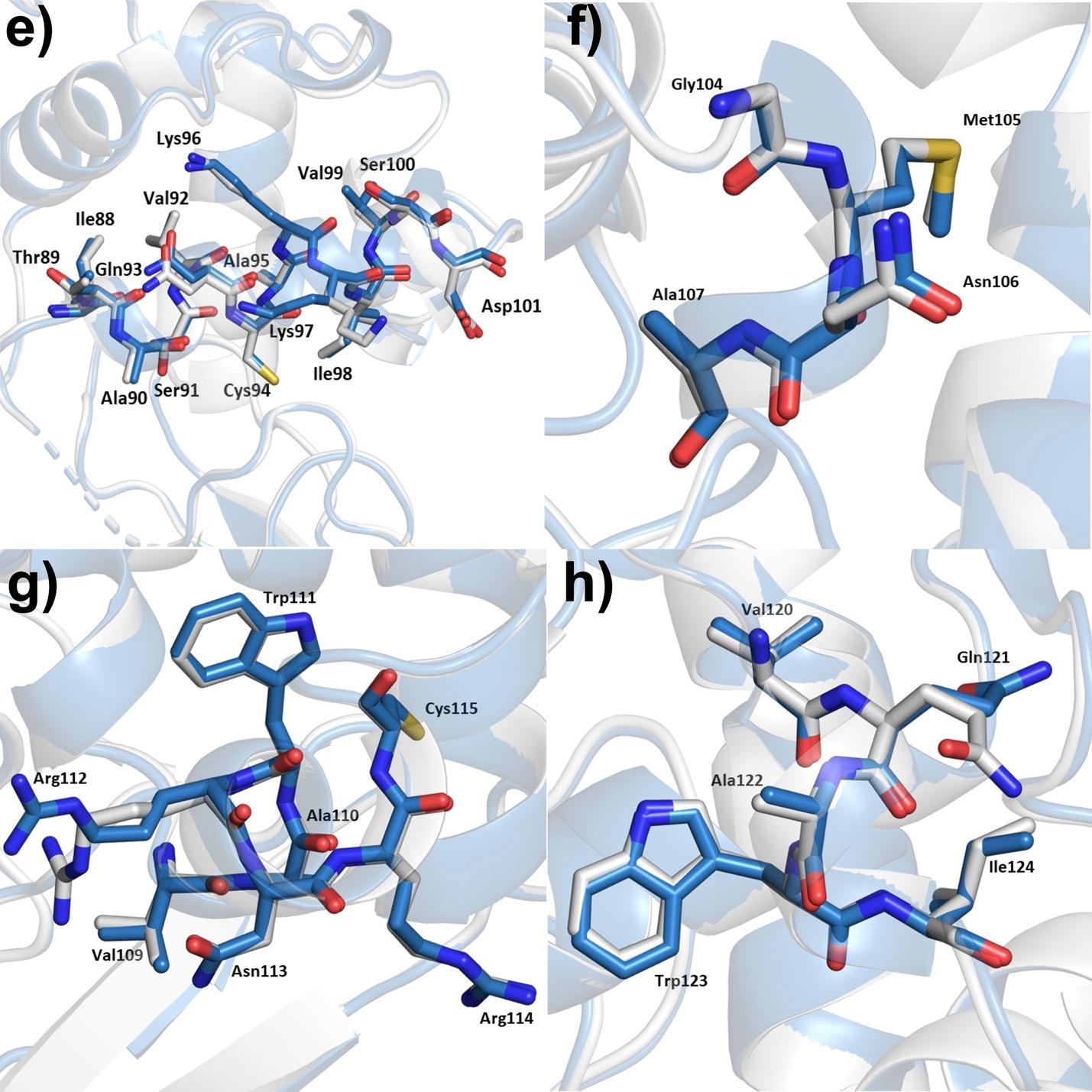
**

**Supplementary Figure 11.** Helix region (5-8) comparisons of ambient temperature lysozyme (skyblue) with cryogenic lysozyme (gray). RMS values are shown in parentheses. **(a)** Helix 5 (*0.100 Å*); **(b)** Helix 6 (*0.101 Å*); **(c)** Helix 7 (*0.115 Å*); **(d)** Helix 8 (*0.087 Å*). Generated with *PyMOL version 2.3* ^27^.

**
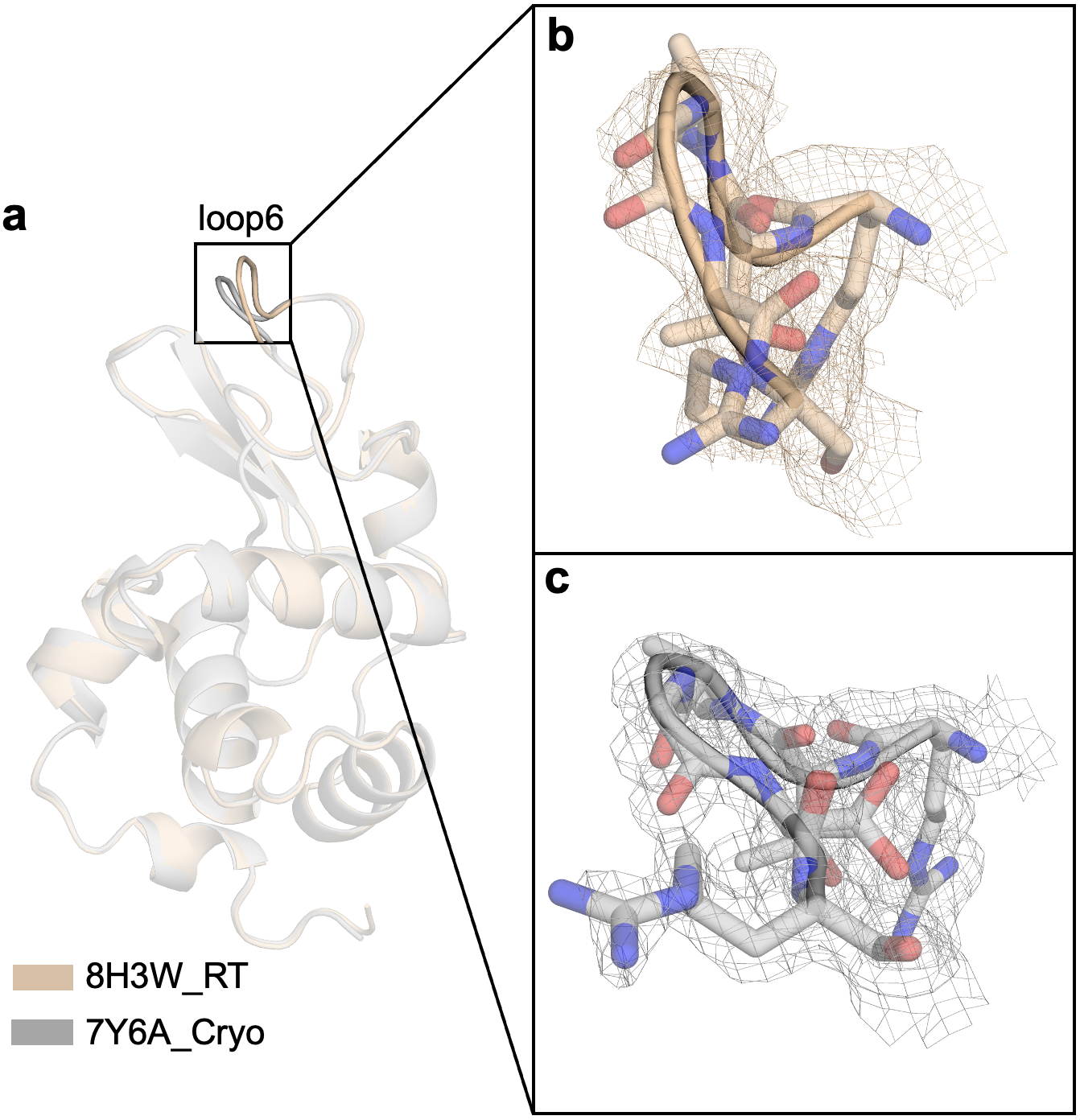
**

**Supplementary Figure 12.** The superposition of room and cryogenic temperature structure of lysozyme. **(a)** The room temperature structure of lysozyme is superposed with the cryogenic temperature structure of lysozyme with an RMSD value of 0.256. **(b)** 2Fo-Fc simulated annealing-omit map for the loop6 of room temperature structure is shown in wheat and contoured at 1.0 σ level. **(c)** 2Fo-Fc simulated annealing-omit map for the loop6 of cryogenic temperature structure is shown in gray and contoured at 1.0 σ level. Generated with *PyMOL version 2.3* ^27^.
